# Liver-Directed Therapy for Neuroendocrine Tumor Metastases in the Era of Peptide Receptor Radionuclide Therapy

**DOI:** 10.1007/s11864-023-01152-6

**Published:** 2023-12-15

**Authors:** Rana Rabei, Nicholas Fidelman

**Affiliations:** Department of Radiology and Biomedical Imaging, University of California San Francisco, 505 Parnassus Avenue, Room M-361, San Francisco, CA 94143 USA

**Keywords:** Neuroendocrine tumor, Liver metastases, Embolization, Chemoembolization, Radioembolization, Ablation

## Abstract

The treatment of neuroendocrine neoplasm (NEN) liver metastases involves a multidisciplinary approach that includes liver-directed therapies (LDT) and systemic treatments, such as peptide receptor radionuclide therapy (PRRT). LDT has demonstrated efficacy in rapidly reducing tumor bulk, improving symptoms, and delaying disease progression. Interventional radiologists should be consulted prior to switching therapy for patients with progressive or symptomatic neuroendocrine tumor liver metastases. Long-term follow-up data on the safety of Yttrium-90 radioembolization before and after PRRT remain limited. Therefore, a more conservative approach may be to preferentially employ transarterial embolization (TAE) or transarterial chemoembolization (TACE) for patients’ somatostatin receptor-avid disease who may be future candidates for PRRT. Notable exceptions where radioembolization may be a preferred treatment strategy may be patients with history of biliary tract instrumentation, asymmetric unilobar disease distribution, and rapidly progressive diffuse liver involvement. Selection of local treatment modality, sequencing, and combination of LDT with systemic therapy require further investigation.

## Introduction

Neuroendocrine neoplasms (NEN) are a rare but complex group of malignancies that arise from the enterochromaffin cells with neuroendocrine differentiation. These tumors can originate in various organs, including the gastrointestinal tract, pancreas, lungs, and thyroid gland. Functional NEN may cause hormonally mediated symptoms such as flushing, diarrhea (carcinoid syndrome), and hypoglycemia, while nonfunctional tumors often present at later stages due to lack of symptom-causing hormone production.

Liver metastases are common in patients with NEN, and they can significantly affect prognosis and quality of life. In fact, up to 80% of patients with NEN will develop liver metastases at some point in their disease course. The treatment of liver metastases from NEN requires a multidisciplinary approach, including surgical resection, liver-directed therapies (LDT), and systemic agents [1].

Liver-directed therapies play a crucial role in the management of patients with unresectable NEN liver metastases by reducing patient’s hormonal and bulk symptom burden, delaying liver failure, diminishing risk of carcinoid heart disease, and potentially improving the efficacy of systemic therapy. Forms of LDT include catheter-based techniques such as transarterial embolization (TAE), transarterial chemoembolization (TACE), and transarterial radioembolization (TARE); ablative therapies such as microwave ablation (MWA) and cryoablation, as well as stereotactic ablative radiotherapy (SABR). In clinical practice, the choice of LDT is made upon consideration of a variety of factors, including size, location, vascularity, and number of the liver metastases, extent of liver replacement by tumor, tumor grade, symptoms, and the patient’s overall health status and comorbidities [2]. Identification of molecular and imaging biomarkers to predict treatment response and toxicity and guide choice of therapy is a promising area of research.

Systemic treatments for NEN include somatostatin analogues, chemotherapy, targeted agents (e.g., everolimus and sunitinib), and peptide receptor radionuclide therapy (PRRT). PRRT involves the systemic administration of radiolabeled somatostatin analogues, which bind to somatostatin receptors expressed on NET cells, delivering a targeted dose of radiation. In a phase III clinical trial (NETTER-1), PRRT has been shown to be effective in controlling tumor growth and improving symptoms in patients with advanced NETs compared to high-dose octreotide. Secondary analysis from NETTER-1 trial has identified higher tumor volume to be associated with worse response to PRRT, making the case for debulking of hepatic lesions prior to initiation of PRRT [3, 4•].

Approval of Lutathera® PRRT by the US Food and Drug Administration in 2018 dramatically changed NEN treatment landscape, and the role of LDT in the treatment of NET liver metastases is being redefined and continuously re-evaluated. Timing of LDT in the course of the disease, sequencing with other therapies, and choice of LDT remain under active investigation.

## Choosing among transarterial liver-directed therapies

LDTs are safe and effective for treatment for NEN liver metastases. These therapies have been shown to reduce tumor bulk, alleviate tumor-related symptoms, and reduce hormone levels. Percutaneous thermal ablation may be applied for treatment of few small tumors (generally three or fewer lesions, < 3 cm in size) as a cytoreductive strategy for all detectable liver lesions or for discordant enlarging lesions in the setting of stability of other disease.

Embolotherapies are used for control of larger lesions and/or multifocal disease. The extent of liver parenchyma replacement by tumor prior to embolotherapy has not been agreed upon. However, it may be reasonable to consider embolization when the extent of liver replacement by tumor is between 10 and 90%. Treating few small scattered lesions with embolization would expose large zones of normal liver parenchyma to ischemic, chemical, or radiation toxicity, potentially contributing to long-term liver dysfunction. Delaying embolization until liver parenchyma replacement is > 10% and/or until the largest target lesion size reaches 3 cm may be a reasonable strategy barring debilitating hormonal symptoms that could be addressed with thermal ablation, TAE, or TACE to the dominant liver lesion(s) expeditiously. Surgical resection (if feasible) may be reasonable to consider in the setting of low-volume multi-focal liver disease. Alternatively, systemic therapies such as somatostatin analogues and targeted agents may be helpful while the “wait and not embolize” strategy is pursued. Embolization in the setting of extensive liver parenchyma replacement by tumor (> 90%) may result in a high risk of irreversible hepatotoxicity due to the limited liver parenchyma reserve [2]. Theoretically, serial transarterial embolization with meticulous post-procedure liver function monitoring may be safer than TACE or TARE for patients with extensive (70–90%) liver parenchyma replacement due to lack of exposure of the remaining healthy parenchyma to cytotoxic chemotherapy or radiation.

Determining the optimal embolotherapy modality among TAE, TACE, and TARE remains a challenge.

A multicenter retrospective study of 155 patients who underwent transarterial therapy for NEN liver metastases found that higher tumor grade and tumor burden correlate with shorter hepatic progression-free survival and overall survival across treatment modalities. Propensity score adjusted multivariate analysis demonstrated TARE to have a higher hazard ratio for overall survival (HR 1.8, *p* = 0.11) without significant difference in hepatic progression-free survival [5].

With wide adoption of PRRT, concerns have been raised about the use of TARE before or after PRRT. Long-term liver toxicity following TARE for treatment of NEN liver metastases has been observed [6•, 7]. The term radioembolization-induced chronic hepatotoxicity (RECHT) refers to clinical signs, symptoms, and laboratory and/or imaging findings of chronic liver disease in the absence of liver tumor progression that may be observed following TARE [7]. Several studies have examined the risk of long-lasting hepatotoxicity following TARE. The prospective RESiN registry, which assessed the outcomes of ^90^Y radioembolization spheres for neuroendocrine liver metastases in 177 patients, found that 7.6% of patients developed grade 3 or worse hepatic toxicity. In various single-center retrospective analyses, hepatotoxicity rates ranged from 14 to 48%, with larger treatment areas associated with higher toxicity [6•, 7, 8]. Radiation oncology concept mandating that whole liver radiation should be avoided in order to prevent radiation induced liver disease (RILD) is currently being applied to radioembolization [9]. Patients with bilobar disease may receive treatment with radioembolization to the predominantly involved liver lobe. The remaining 2–3 liver segments are spared radiation treatment and may be treated with another modality such as embolization, TACE, or thermal ablation. The use of low-dose corticosteroids and ursodiol for 2 months following lobar TARE as well as avoidance of whole liver TARE has been shown to reduce the risk of radioembolization-induced liver disease [10].

Studies evaluating the safety of sequencing TARE and PRRT have been mostly retrospective, with small sample sizes and limited follow-up periods [11]. Nevertheless, available evidence suggests that TARE can be used safely before or after PRRT.

The use of TAE and TACE rather than TARE has been advocated in the era of PRRT since these modalities do not involve deposition of radiation in the liver tissue. However, data supporting this treatment selection strategy are lacking. While one study reported a high incidence of PRRT-induced hepatotoxicity in heavily pretreated patients with liver-dominant disease, with ascites developing in 59% of the PRRT group compared to 6.6% in the unexposed group (*P* < 0.001), other studies have not found a significant increase in the risk of liver toxicity among patients who received locoregional therapy before or after PRRT [12–15].

Patients with history of biliary tract instrumentation (e.g., sphincterotomy, biliary stent and/or drain, and biliary-enteric anastomosis) are at an increased risk for infection following LDT due to biliary tract colonization. Risk of major hepatobiliary infection, such as liver abscess formation and cholangitis, appears to be significantly lower for TARE ranging around 2%, compared to TACE which carries a 23–86% risk of hepatobiliary infection based on retrospective reports [16].

RETNET randomized phase II trial is an ongoing multicenter prospective trial, which aims to assess tumor response, hepatic progression-free survival, and overall survival in participants treated with TAE, conventional, and drug-eluting bead (DEB) TACE (NCT02724540). The first safety report from the trial demonstrated a high rate of adverse events in the DEB-TACE arm, resulting in its closure [17, 18]. RETNET results may help clinicians to make informed decisions about TAE and conventional TACE for NEN liver metastases.

Relative merits of conventional TACE or TAE versus TARE depending on commonly encountered clinical scenarios are summarized in Table 1. TAE or cTACE may be a preferred treatment strategy for patients who may benefit from PRRT in the future. When one or few lesions are enlarging in the setting of otherwise stable disease, percutaneous thermal ablation or Yttrium-90 radiation segmentectomy could be considered. Radiation segmentectomy is a technique that involves administration of ablative radiation dose (> 200 Gy) to no more than two Cuinaud liver segments, and has been widely adopted for treatment of unresectable localized hepatocellular carcinoma [19, 20]. Data behind using radiation segmentectomy for metastatic disease to the liver have been limited [21]. Ablative radiation dose would be expected to result in irreversible damage to the target liver segment, and wide application of this strategy for patients with oligo-progressive NETLM may conflict with the concept of long-term liver health preservation. Lobar radioembolization may be favored for patients with asymmetric bulky unilobar disease distribution or in the rapidly progressive disease due to concern of out-of treatment field progression during sequential TACE or TAE.

**Table 1:** Suggested guideline for selection of liver-directed therapy for well-differentiated NET liver metastases

Clinical scenario	cTACE/TAE	TARE
DOTA-avid liver only/dominant, progressive disease on SSA	+ + +	−
DOTA-avid liver only/dominant, radiographically stable, but severe hormonal symptoms or high 5-HIAA	+ + +	−
DOTA-avid diffuse or liver-dominant, one or few enlarging liver lesions, rest stable on current therapy	+ + + (also ablation)	+ (rad seg)
DOTA-avid diffuse or liver-dominant, but one or few liver lesions with low uptake	+ + + (also ablation)	+ (rad seg)
DOTA-avid, bulky liver lesion(s)	+ + +	+
Progression after PRRT	+ + +	−
Not DOTA-avid	+ + +	+
One lobe predominant bulky disease	+ + +	+ + +
Rapid bilobar progression*	+	+ + +
History of biliary instrumentation (Whipple, biliary stent, drain)	−	+ + +

+  +  + , preferred treatment strategy

+ , reasonable treatment strategy

− , not recommended

*Rad seg*, radiation segmentectomy. Radiation segmentectomy may be considered for patients with one or more lesions within a 1–2 Cuinaud liver segments. Delivery of ablative radiation dose is expected to cause irreversible injury to the treated liver segment. Therefore, this technique should be used judiciously with the goal of preservation of liver reserve

^*^Patients with rapid bilobar liver disease progression may benefit from combination of TARE and radiosensitizers capecitabine and temozolomide (21)

## Combination of LDT and systemic therapies

TARE may have a synergistic response with radiosensitizing systemic chemotherapy. Capecitabine-temozolomide (CapTem) is an effective oral chemotherapy regimen for NEN, and both drugs are radiosensitizers. A recent study showed that combination of CapTem and TARE resulted in durable control of grade 2 NEN liver metastases for substantially longer than expected for either regimen alone with median PFS of 36 months (95% CI, 25–45 months). Overall survival was 41 months (95% CI, 24–87 months) from initiation of CapTemY90 therapy and 130 months (95% CI, 56–172 months) from initial NEN diagnosis [22•].

Over the past decade, immunotherapy has dramatically improved the outlook for many solid tumors; however, the effectiveness of immune checkpoint inhibitors (ICIs) in treating well-differentiated NENs has been limited. Well-differentiated NENs generally have slow growth rates and low tumor mutation burden. Studies have suggested that treatment with ICIs has low response rates for these patients [23]. However, the intensity of anti-tumor immune response may be enhanced by LDT. Radiofrequency ablation, cryoablation, TAE, TACE, and TARE have been evaluated in this context in other tumor types with promising results [24]. Abscopal effect, where localized treatment of a tumor causes regression of target and non-target tumors, has been reported after radiotherapy in combination with ICIs [25]. A pilot clinical trial is currently underway to evaluate whether combining PDL-1 inhibitor pembrolizumab with LDT can result in abscopal effects for patients with well-differentiated NEN (NCT03457948) [26].

## Prevention of carcinoid crisis

Carcinoid crisis is a serious and rare complication of liver directed therapy for NEN liver metastases. This condition arises from release of vasoactive substances, such as serotonin and histamine, from damaged tumor cells. The resulting symptoms can be severe and potentially life-threatening, including hypertension or hypotension, tachycardia or bradycardia, bronchospasm, flushing, and diarrhea. Immediate recognition and treatment are crucial to prevent respiratory failure, cardiac arrest, and death. Therefore, close monitoring is essential for patients undergoing liver resection or LDT to detect and treat any signs of carcinoid crisis promptly. A retrospective study of patients undergoing liver-directed therapy for functional mid-gut NEN metastases reported rates of carcinoid crisis in 22% of patients undergoing transarterial therapy and 42% with liver resection and/or surgical ablation [27].

In our practice, patients with moderate or severe carcinoid syndrome at baseline or with insulinoma are routinely scheduled for LDT procedures with anesthesia support. To mitigate the risk of carcinoid crisis during LDT, we have implemented a pre-treatment regimen of H1 or H2 receptor blockers, subcutaneous short-acting octreotide at the dose of 150 mcg, and intravenous octreotide infusion at the rate of 150–300 mcg/h. Intraprocedural hemodynamic instability may be treated with intravenous boluses of octreotide 500 mcg administered every 5–10 min. Concurrently, intravenous vasopressors, antihistamines, and fluids may be administered as needed, excluding epinephrine and norepinephrine, which may precipitate or exacerbate carcinoid crisis. Depending on post-procedure carcinoid syndrome symptom severity, intravenous octreotide (150 mcg/h) may be continued for 2 to 24 h or longer. Additional subcutaneous short-acting octreotide at the dose of 100–200 mcg every 8 h as well as H1 and H2 blockers may be administered for post-procedure mitigation of carcinoid syndrome symptoms.

## Intra-arterial PRRT

Intra-arterial (IA) administration of PRRT is currently under investigation. This technique involves the direct injection of PRRT into the hepatic artery, which is the main blood supply to NEN liver metastases. IA PRRT is hypothesized to increase the absorbed radiation dose in liver metastases while reducing radiation exposure to the normal hepatic parenchyma, kidneys, and bone marrow when compared with systemic 177Lu or ^90^Y DOTATATE administration. Preliminary work using ^68^ Ga-DOTATOC used as a surrogate marker of therapeutic somatostatin analogues demonstrated an increased hepatic tumor uptake with intra-arterial compared to intravenous administration [28]. Pilot trials that evaluated IA and IV PRRT showed favorable clinical outcomes with multiple intra-arterial administrations [29, 30]. The LUTIA trial (NCT03590119) is an ongoing multicenter, interventional, randomized phase 2 clinical trial that aims to investigate whether IA administration of 177Lu-DOTATATE results in a higher radiation dose to liver metastases compared to IV administration [31].

## LDT innovations: histotripsy

Histotripsy is a novel non-invasive approach to liver directed therapy, which uses high-power and low-frequency ultrasound energy for tissue destruction. Compared to conventional ablative technologies such as MWA and cryoablation, histotripsy offers a needle-less approach to cancer treatment, precise targeting with ultrasound visualization, and real-time feedback from ultrasound imaging for pre- and peri-operative treatment planning and monitoring. The first human trial of histotripsy, the Theresa trial, found it to be well-tolerated, achieving its primary endpoint of technical success in all 8 enrolled patients with primary or secondary liver tumors [32•]. Building on this success, the ongoing HistoSonics System for Treatment of Primary and Metastatic Liver Tumors Using Histotripsy trial (NCT04573881) aims to evaluate the safety and efficacy of histotripsy in a larger patient population with primary and metastatic liver tumors, potentially providing a new non-invasive treatment option for patients with metastatic NEN [33].

## Future directions: treatment selection in the era of molecular profiling

As precision medicine becomes increasingly prevalent, molecular biomarkers are being used to predict disease prognosis and treatment response. Advances in molecular techniques have led to the identification of prognostic biomarkers for pancreatic NEN, such as loss of ATRX/DAXX and the presence of ALT, which have been shown to correlate with adverse pathological features in nonfunctional pancreatic NEN, such as larger tumor size, higher WHO grade, lymphovascular and perineural invasion, and distant metastases more rapid recurrence on follow-up [34]. Presence of ATRX/DAXX mutations has been associated with worse hepatic PFS and time to hepatic progression after TAE, but conferred a better treatment response for patients treated with capecitabine and temozolomide [35].

PPQ blood-based transcriptome assay has been validated to measure PRRT sensitivity prediction by evaluating genes associated with growth factor signaling and metabolism of NEN, along with tumor grade. While PRRT-specific radiation sensitivity assays are yet to be validated, preliminary studies suggest that somatic mutations in chromatin remodeling, DNA damage/repair, and apoptosis genes may serve as viable targets for evaluation. These observations may also prove to be relevant in prediction of response and toxicity after TARE. The prediction of treatment-related toxicities is also crucial in neuroendocrine tumor treatment, requiring the assessment of germline single nucleotide polymorphisms in a series of genes that are proven radiation sensitivity intrinsic factors. Lastly, epigenetic evaluation may offer alternative molecular strategies for either sensitivity or toxicity prediction [36].

## Conclusion

Treatment of NEN liver metastases requires a multidisciplinary approach that relies upon LDT and systemic therapy. LDT is effective in reducing disease bulk, improving hormone-mediated symptoms, and delaying disease progression. Therefore, LDT should be considered when switching treatments at the time of disease progression. Long-term follow-up data on the safety of Yttrium-90 radioembolization before and after PRRT remain limited. Therefore, a more conservative approach may be to preferentially employ TAE or TACE for patients with somatostatin receptor-avid disease who may be future candidates for PRRT. Notable exceptions where radioembolization may be a preferred treatment strategy may be patients with history of biliary tract instrumentation, asymmetric unilobar disease distribution, and rapidly progressive diffuse liver involvement. Further research is needed to determine the optimal selection, sequencing, and combination of LDT with systemic therapy.
